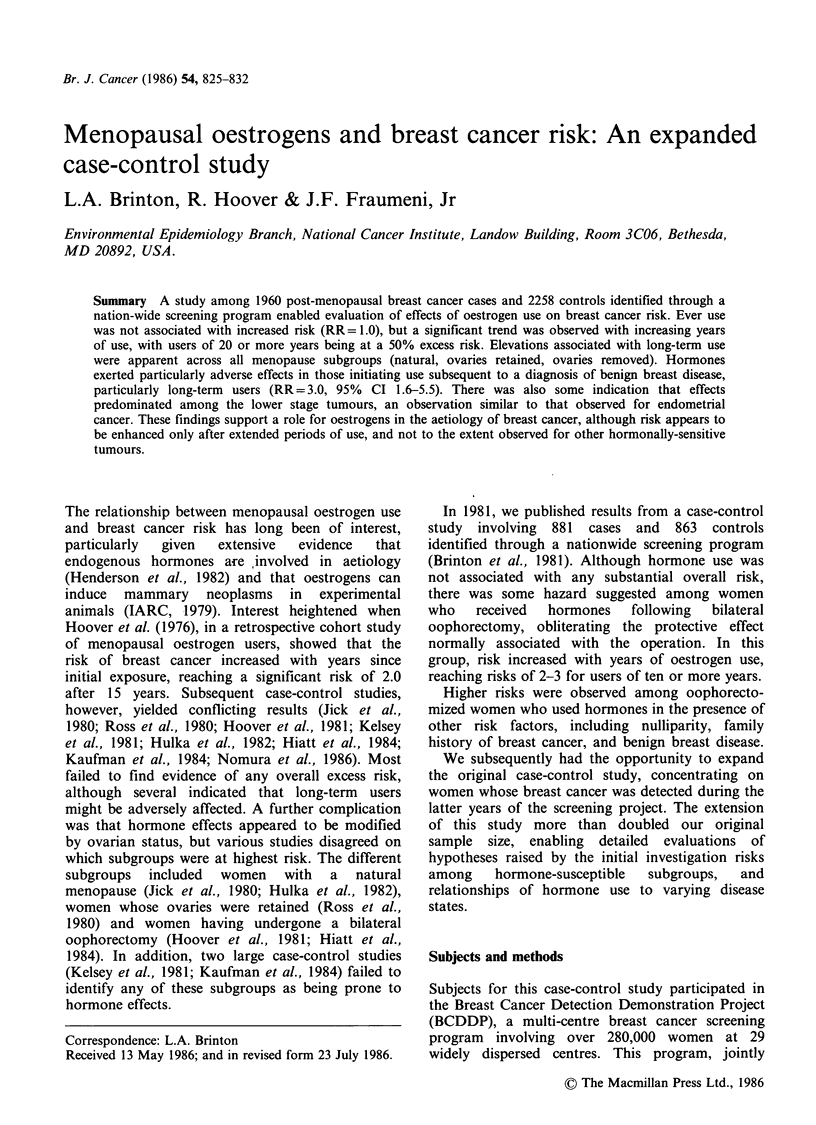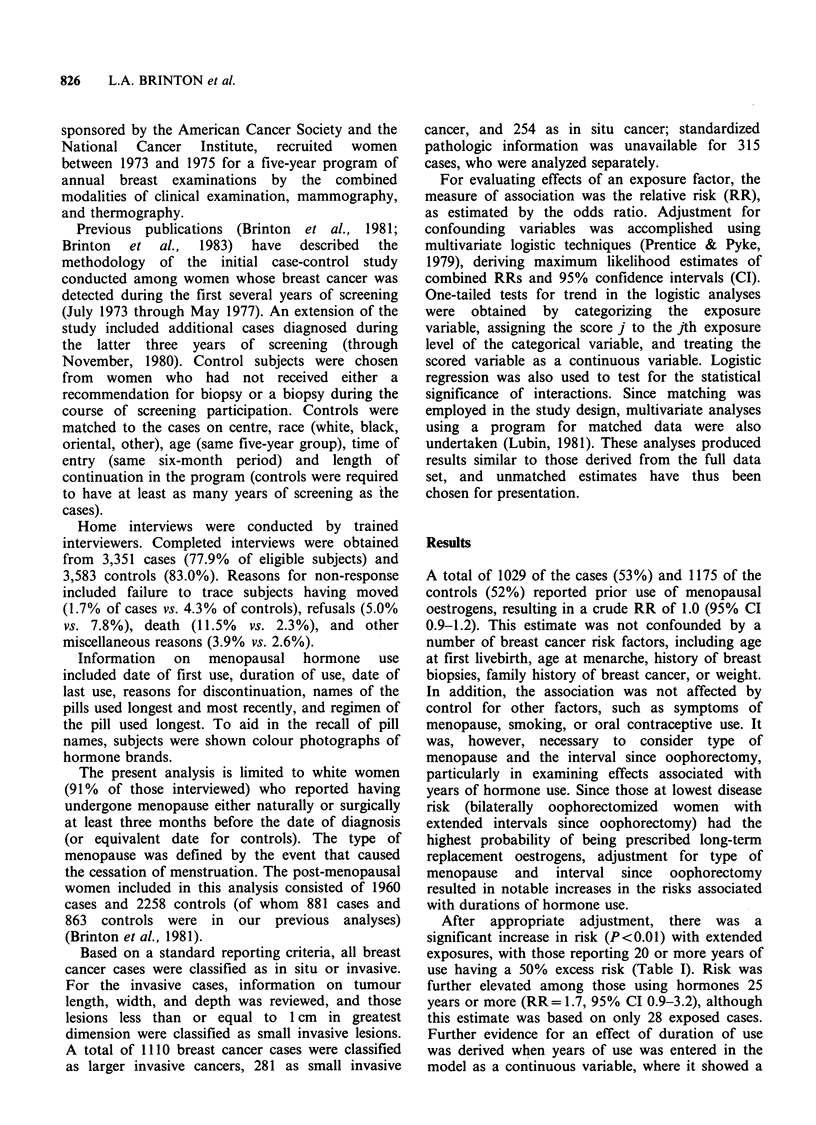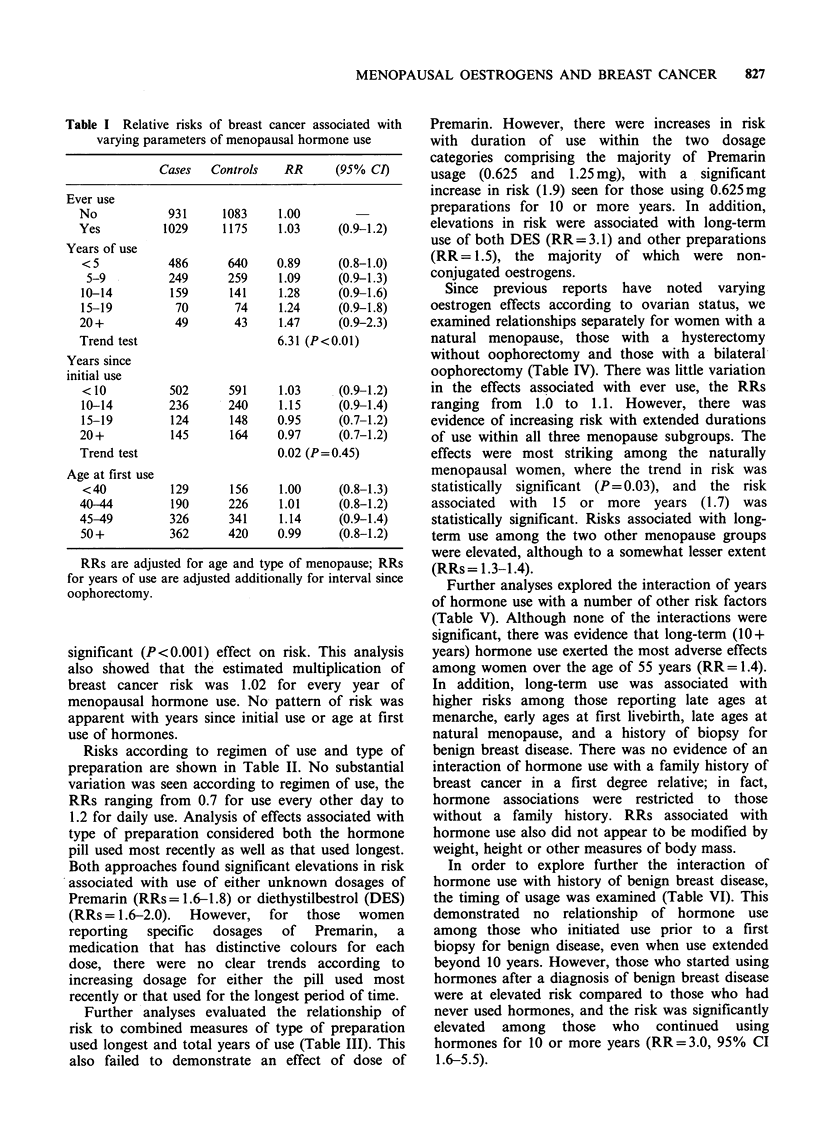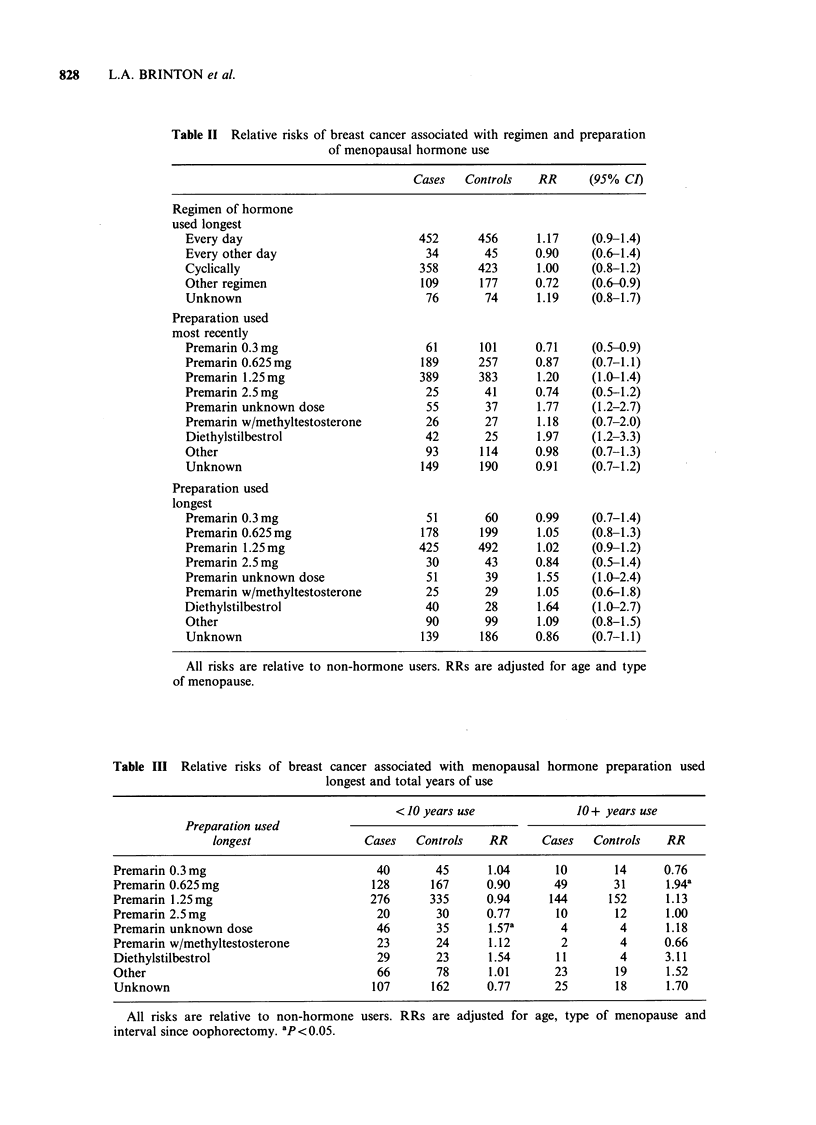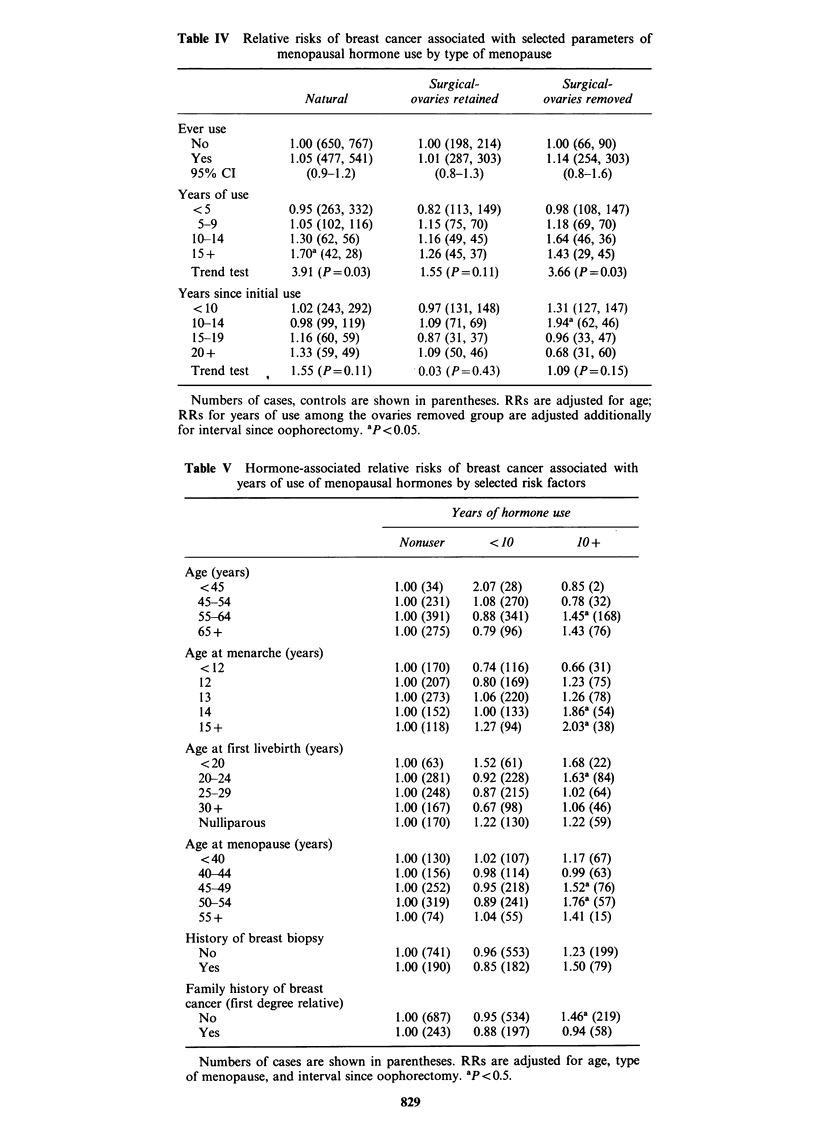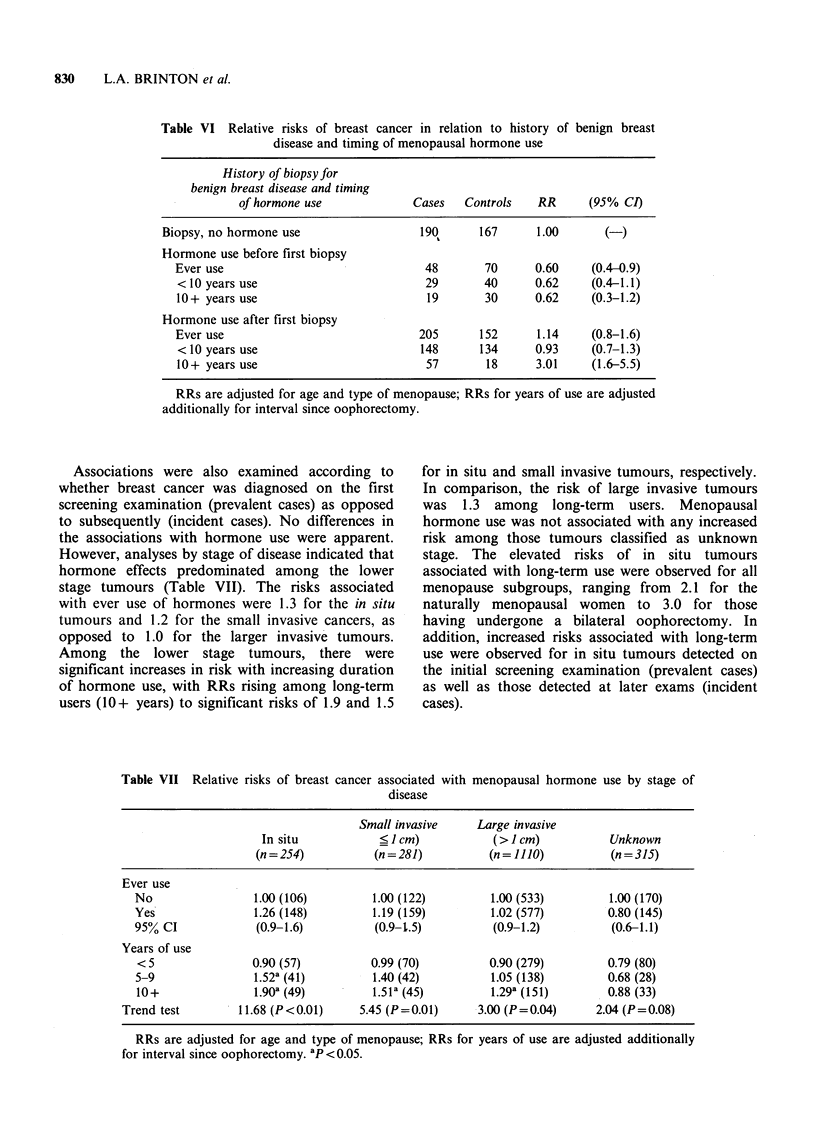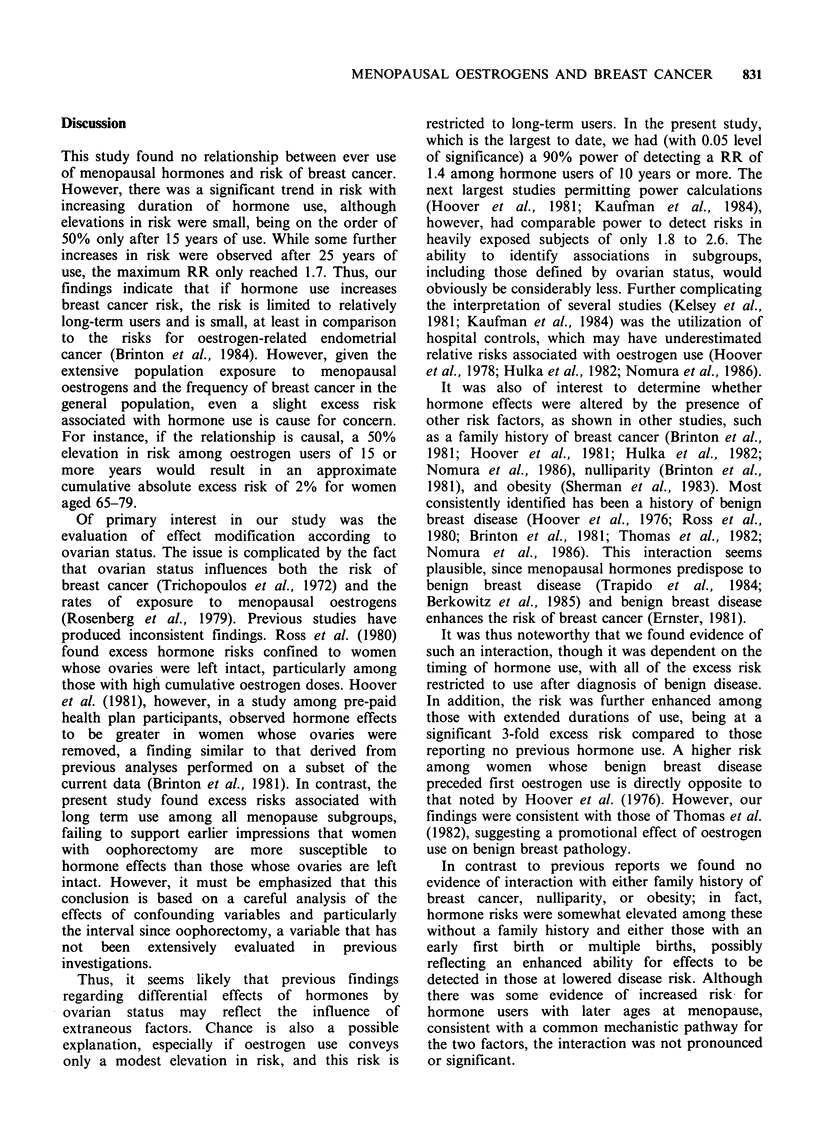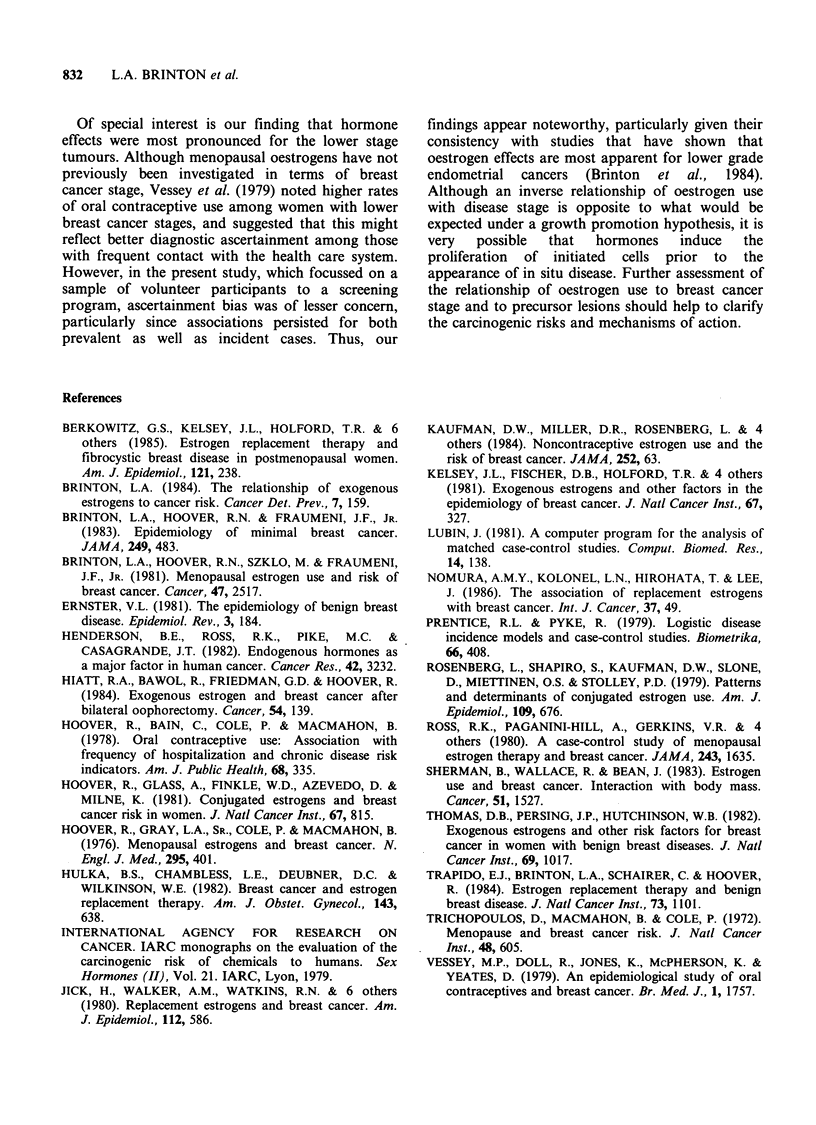# Menopausal oestrogens and breast cancer risk: an expanded case-control study.

**DOI:** 10.1038/bjc.1986.246

**Published:** 1986-11

**Authors:** L. A. Brinton, R. Hoover, J. F. Fraumeni

## Abstract

A study among 1960 post-menopausal breast cancer cases and 2258 controls identified through a nation-wide screening program enabled evaluation of effects of oestrogen use on breast cancer risk. Ever use was not associated with increased risk (RR = 1.0), but a significant trend was observed with increasing years of use, with users of 20 or more years being at a 50% excess risk. Elevations associated with long-term use were apparent across all menopause subgroups (natural, ovaries retained, ovaries removed). Hormones exerted particularly adverse effects in those initiating use subsequent to a diagnosis of benign breast disease, particularly long-term users (RR = 3.0, 95% CI 1.6-5.5). There was also some indication that effects predominated among the lower stage tumours, an observation similar to that observed for endometrial cancer. These findings support a role for oestrogens in the aetiology of breast cancer, although risk appears to be enhanced only after extended periods of use, and not to the extent observed for other hormonally-sensitive tumours.


*Br. J. Cancer* (1986) **54**, 825–832

### Menopausal oestrogens and breast cancer risk: An expanded case-control study

L.A. Brinton, R. Hoover & J.F. Fraumeni, Jr

*Environmental Epidemiology Branch, National Cancer Institute, Landow Building, Room 3C06, Bethesda, MD 20892, USA.*

**Summary** A study among 1960 post-menopausal breast cancer cases and 2258 controls identified through a nation-wide screening program enabled evaluation of effects of oestrogen use on breast cancer risk. Ever use was not associated with increased risk (RR = 1.0), but a significant trend was observed with increasing years of use, with users of 20 or more years being at a 50% excess risk. Elevations associated with long-term use were apparent across all menopause subgroups (natural, ovaries retained, ovaries removed). Hormones exerted particularly adverse effects in those initiating use subsequent to a diagnosis of benign breast disease, particularly long-term users (RR = 3.0, 95% CI 1.6–5.5). There was also some indication that effects predominated among the lower stage tumours, an observation similar to that observed for endometrial cancer. These findings support a role for oestrogens in the aetiology of breast cancer, although risk appears to be enhanced only after extended periods of use, and not to the extent observed for other hormonally-sensitive tumours.

The relationship between menopausal oestrogen use and breast cancer risk has long been of interest, particularly given extensive evidence that endogenous hormones are involved in aetiology (Henderson *et al.*, 1982) and that oestrogens can induce mammary neoplasms in experimental animals (IARC, 1979). Interest heightened when Hoover *et al.* (1976), in a retrospective cohort study of menopausal oestrogen users, showed that the risk of breast cancer increased with years since initial exposure, reaching a significant risk of 2.0 after 15 years. Subsequent case-control studies, however, yielded conflicting results (Jick *et al.*, 1980; Ross *et al.*, 1980; Hoover *et al.*, 1981; Kelsey *et al.*, 1981; Hulka *et al.*, 1982; Hiatt *et al.*, 1984; Kaufman *et al.*, 1984; Nomura *et al.*, 1986). Most failed to find evidence of any overall excess risk, although several indicated that long-term users might be adversely affected. A further complication was that hormone effects appeared to be modified by ovarian status, but various studies disagreed on which subgroups were at highest risk. The different subgroups included women with a natural menopause (Jick *et al.*, 1980; Hulka *et al.*, 1982), women whose ovaries were retained (Ross *et al.*, 1980) and women having undergone a bilateral oophorectomy (Hoover *et al.*, 1981; Hiatt *et al.*, 1984). In addition, two large case-control studies (Kelsey *et al.*, 1981; Kaufman *et al.*, 1984) failed to identify any of these subgroups as being prone to hormone effects.

Correspondence: L.A. Brinton
Received 13 May 1986; and in revised form 23 July 1986.

In 1981, we published results from a case-control study involving 881 cases and 863 controls identified through a nationwide screening program (Brinton *et al.*, 1981). Although hormone use was not associated with any substantial overall risk, there was some hazard suggested among women who received hormones following bilateral oophorectomy, obliterating the protective effect normally associated with the operation. In this group, risk increased with years of oestrogen use, reaching risks of 2–3 for users of ten or more years.

Higher risks were observed among oophorectomized women who used hormones in the presence of other risk factors, including nulliparity, family history of breast cancer, and benign breast disease.

We subsequently had the opportunity to expand the original case-control study, concentrating on women whose breast cancer was detected during the latter years of the screening project. The extension of this study more than doubled our original sample size, enabling detailed evaluations of hypotheses raised by the initial investigation risks among hormone-susceptible subgroups, and relationships of hormone use to varying disease states.

### Subjects and methods

Subjects for this case-control study participated in the Breast Cancer Detection Demonstration Project (BCDDP), a multi-centre breast cancer screening program involving over 280,000 women at 29 widely dispersed centres. This program, jointly

© The Macmillan Press Ltd., 1986

sponsored by the American Cancer Society and the National Cancer Institute, recruited women between 1973 and 1975 for a five-year program of annual breast examinations by the combined modalities of clinical examination, mammography, and thermography.

Previous publications (Brinton *et al.*, 1981; Brinton *et al.*, 1983) have described the methodology of the initial case-control study conducted among women whose breast cancer was detected during the first several years of screening (July 1973 through May 1977). An extension of the study included additional cases diagnosed during the latter three years of screening (through November, 1980). Control subjects were chosen from women who had not received either a recommendation for biopsy or a biopsy during the course of screening participation. Controls were matched to the cases on centre, race (white, black, oriental, other), age (same five-year group), time of entry (same six-month period) and length of continuation in the program (controls were required to have at least as many years of screening as the cases).

Home interviews were conducted by trained interviewers. Completed interviews were obtained from 3,351 cases (77.9% of eligible subjects) and 3,583 controls (83.0%). Reasons for non-response included failure to trace subjects having moved (1.7% of cases *vs.* 4.3% of controls), refusals (5.0% *vs.* 7.8%), death (11.5% *vs.* 2.3%), and other miscellaneous reasons (3.9% *vs.* 2.6%).

Information on menopausal hormone use included date of first use, duration of use, date of last use, reasons for discontinuation, names of the pills used longest and most recently, and regimen of the pill used longest. To aid in the recall of pill names, subjects were shown colour photographs of hormone brands.

The present analysis is limited to white women (91% of those interviewed) who reported having undergone menopause either naturally or surgically at least three months before the date of diagnosis (or equivalent date for controls). The type of menopause was defined by the event that caused the cessation of menstruation. The post-menopausal women included in this analysis consisted of 1960 cases and 2258 controls (of whom 881 cases and 863 controls were in our previous analyses) (Brinton *et al.*, 1981).

Based on a standard reporting criteria, all breast cancer cases were classified as in situ or invasive. For the invasive cases, information on tumour length, width, and depth was reviewed, and those lesions less than or equal to 1 cm in greatest dimension were classified as small invasive lesions. A total of 1110 breast cancer cases were classified as larger invasive cancers, 281 as small invasive cancer, and 254 as in situ cancer; standardized pathologic information was unavailable for 315 cases, who were analyzed separately.

For evaluating effects of an exposure factor, the measure of association was the relative risk (RR), as estimated by the odds ratio. Adjustment for confounding variables was accomplished using multivariate logistic techniques (Prentice & Pyke, 1979), deriving maximum likelihood estimates of combined RRs and 95% confidence intervals (CI). One-tailed tests for trend in the logistic analyses were obtained by categorizing the exposure variable, assigning the score $j$ to the $j$th exposure level of the categorical variable, and treating the scored variable as a continuous variable. Logistic regression was also used to test for the statistical significance of interactions. Since matching was employed in the study design, multivariate analyses using a program for matched data were also undertaken (Lubin, 1981). These analyses produced results similar to those derived from the full data set, and unmatched estimates have thus been chosen for presentation.

### Results

A total of 1029 of the cases (53%) and 1175 of the controls (52%) reported prior use of menopausal oestrogens, resulting in a crude RR of 1.0 (95% CI 0.9–1.2). This estimate was not confounded by a number of breast cancer risk factors, including age at first livebirth, age at menarche, history of breast biopsies, family history of breast cancer, or weight. In addition, the association was not affected by control for other factors, such as symptoms of menopause, smoking, or oral contraceptive use. It was, however, necessary to consider type of menopause and the interval since oophorectomy, particularly in examining effects associated with years of hormone use. Since those at lowest disease risk (bilaterally oophorectomized women with extended intervals since oophorectomy) had the highest probability of being prescribed long-term replacement oestrogens, adjustment for type of menopause and interval since oophorectomy resulted in notable increases in the risks associated with durations of hormone use.

After appropriate adjustment, there was a significant increase in risk ($P<0.01$) with extended exposures, with those reporting 20 or more years of use having a 50% excess risk (Table I). Risk was further elevated among those using hormones 25 years or more (RR = 1.7, 95% CI 0.9–3.2), although this estimate was based on only 28 exposed cases. Further evidence for an effect of duration of use was derived when years of use was entered in the model as a continuous variable, where it showed a

**Table I** Relative risks of breast cancer associated with varying parameters of menopausal hormone use

| | *Cases* | *Controls* | *RR* | *(95% CI)* |
|---|---|---|---|---|
| Ever use | | | | |
| No | 931 | 1083 | 1.00 | — |
| Yes | 1029 | 1175 | 1.03 | (0.9–1.2) |
| Years of use | | | | |
| <5 | 486 | 640 | 0.89 | (0.8–1.0) |
| 5–9 | 249 | 259 | 1.09 | (0.9–1.3) |
| 10–14 | 159 | 141 | 1.28 | (0.9–1.6) |
| 15–19 | 70 | 74 | 1.24 | (0.9–1.8) |
| 20+ | 49 | 43 | 1.47 | (0.9–2.3) |
| Trend test | | | 6.31 ($P<0.01$) | |
| Years since initial use | | | | |
| <10 | 502 | 591 | 1.03 | (0.9–1.2) |
| 10–14 | 236 | 240 | 1.15 | (0.9–1.4) |
| 15–19 | 124 | 148 | 0.95 | (0.7–1.2) |
| 20+ | 145 | 164 | 0.97 | (0.7–1.2) |
| Trend test | | | 0.02 ($P=0.45$) | |
| Age at first use | | | | |
| <40 | 129 | 156 | 1.00 | (0.8–1.3) |
| 40–44 | 190 | 226 | 1.01 | (0.8–1.2) |
| 45–49 | 326 | 341 | 1.14 | (0.9–1.4) |
| 50+ | 362 | 420 | 0.99 | (0.8–1.2) |

RRs are adjusted for age and type of menopause; RRs for years of use are adjusted additionally for interval since oophorectomy.

significant ($P<0.001$) effect on risk. This analysis also showed that the estimated multiplication of breast cancer risk was 1.02 for every year of menopausal hormone use. No pattern of risk was apparent with years since initial use or age at first use of hormones.

Risks according to regimen of use and type of preparation are shown in Table II. No substantial variation was seen according to regimen of use, the RRs ranging from 0.7 for use every other day to 1.2 for daily use. Analysis of effects associated with type of preparation considered both the hormone pill used most recently as well as that used longest. Both approaches found significant elevations in risk associated with use of either unknown dosages of Premarin (RRs = 1.6–1.8) or diethystilbestrol (DES) (RRs = 1.6–2.0). However, for those women reporting specific dosages of Premarin, a medication that has distinctive colours for each dose, there were no clear trends according to increasing dosage for either the pill used most recently or that used for the longest period of time.

Further analyses evaluated the relationship of risk to combined measures of type of preparation used longest and total years of use (Table III). This also failed to demonstrate an effect of dose of Premarin. However, there were increases in risk with duration of use within the two dosage categories comprising the majority of Premarin usage (0.625 and 1.25 mg), with a significant increase in risk (1.9) seen for those using 0.625 mg preparations for 10 or more years. In addition, elevations in risk were associated with long-term use of both DES (RR = 3.1) and other preparations (RR = 1.5), the majority of which were non-conjugated oestrogens.

Since previous reports have noted varying oestrogen effects according to ovarian status, we examined relationships separately for women with a natural menopause, those with a hysterectomy without oophorectomy and those with a bilateral oophorectomy (Table IV). There was little variation in the effects associated with ever use, the RRs ranging from 1.0 to 1.1. However, there was evidence of increasing risk with extended durations of use within all three menopause subgroups. The effects were most striking among the naturally menopausal women, where the trend in risk was statistically significant ($P=0.03$), and the risk associated with 15 or more years (1.7) was statistically significant. Risks associated with long-term use among the two other menopause groups were elevated, although to a somewhat lesser extent (RRs = 1.3–1.4).

Further analyses explored the interaction of years of hormone use with a number of other risk factors (Table V). Although none of the interactions were significant, there was evidence that long-term (10+ years) hormone use exerted the most adverse effects among women over the age of 55 years (RR = 1.4). In addition, long-term use was associated with higher risks among those reporting late ages at menarche, early ages at first livebirth, late ages at natural menopause, and a history of biopsy for benign breast disease. There was no evidence of an interaction of hormone use with a family history of breast cancer in a first degree relative; in fact, hormone associations were restricted to those without a family history. RRs associated with hormone use also did not appear to be modified by weight, height or other measures of body mass.

In order to explore further the interaction of hormone use with history of benign breast disease, the timing of usage was examined (Table VI). This demonstrated no relationship of hormone use among those who initiated use prior to a first biopsy for benign disease, even when use extended beyond 10 years. However, those who started using hormones after a diagnosis of benign breast disease were at elevated risk compared to those who had never used hormones, and the risk was significantly elevated among those who continued using hormones for 10 or more years (RR = 3.0, 95% CI 1.6–5.5).

**Table II** Relative risks of breast cancer associated with regimen and preparation of menopausal hormone use

| | *Cases* | *Controls* | *RR* | *(95% CI)* |
|---|---|---|---|---|
| Regimen of hormone used longest | | | | |
| Every day | 452 | 456 | 1.17 | (0.9–1.4) |
| Every other day | 34 | 45 | 0.90 | (0.6–1.4) |
| Cyclically | 358 | 423 | 1.00 | (0.8–1.2) |
| Other regimen | 109 | 177 | 0.72 | (0.6–0.9) |
| Unknown | 76 | 74 | 1.19 | (0.8–1.7) |
| Preparation used most recently | | | | |
| Premarin 0.3 mg | 61 | 101 | 0.71 | (0.5–0.9) |
| Premarin 0.625 mg | 189 | 257 | 0.87 | (0.7–1.1) |
| Premarin 1.25 mg | 389 | 383 | 1.20 | (1.0–1.4) |
| Premarin 2.5 mg | 25 | 41 | 0.74 | (0.5–1.2) |
| Premarin unknown dose | 55 | 37 | 1.77 | (1.2–2.7) |
| Premarin w/methyltestosterone | 26 | 27 | 1.18 | (0.7–2.0) |
| Diethylstilbestrol | 42 | 25 | 1.97 | (1.2–3.3) |
| Other | 93 | 114 | 0.98 | (0.7–1.3) |
| Unknown | 149 | 190 | 0.91 | (0.7–1.2) |
| Preparation used longest | | | | |
| Premarin 0.3 mg | 51 | 60 | 0.99 | (0.7–1.4) |
| Premarin 0.625 mg | 178 | 199 | 1.05 | (0.8–1.3) |
| Premarin 1.25 mg | 425 | 492 | 1.02 | (0.9–1.2) |
| Premarin 2.5 mg | 30 | 43 | 0.84 | (0.5–1.4) |
| Premarin unknown dose | 51 | 39 | 1.55 | (1.0–2.4) |
| Premarin w/methyltestosterone | 25 | 29 | 1.05 | (0.6–1.8) |
| Diethylstilbestrol | 40 | 28 | 1.64 | (1.0–2.7) |
| Other | 90 | 99 | 1.09 | (0.8–1.5) |
| Unknown | 139 | 186 | 0.86 | (0.7–1.1) |

All risks are relative to non-hormone users. RRs are adjusted for age and type of menopause.

**Table III** Relative risks of breast cancer associated with menopausal hormone preparation used longest and total years of use

| *Preparation used longest* | *< 10 years use* | | | *10+ years use* | | |
|---|---|---|---|---|---|---|
| | *Cases* | *Controls* | *RR* | *Cases* | *Controls* | *RR* |
| Premarin 0.3 mg | 40 | 45 | 1.04 | 10 | 14 | 0.76 |
| Premarin 0.625 mg | 128 | 167 | 0.90 | 49 | 31 | 1.94[a] |
| Premarin 1.25 mg | 276 | 335 | 0.94 | 144 | 152 | 1.13 |
| Premarin 2.5 mg | 20 | 30 | 0.77 | 10 | 12 | 1.00 |
| Premarin unknown dose | 46 | 35 | 1.57[a] | 4 | 4 | 1.18 |
| Premarin w/methyltestosterone | 23 | 24 | 1.12 | 2 | 4 | 0.66 |
| Diethylstilbestrol | 29 | 23 | 1.54 | 11 | 4 | 3.11 |
| Other | 66 | 78 | 1.01 | 23 | 19 | 1.52 |
| Unknown | 107 | 162 | 0.77 | 25 | 18 | 1.70 |

All risks are relative to non-hormone users. RRs are adjusted for age, type of menopause and interval since oophorectomy. [a]$P < 0.05$.

**Table IV** Relative risks of breast cancer associated with selected parameters of menopausal hormone use by type of menopause

| | *Natural* | *Surgical-ovaries retained* | *Surgical-ovaries removed* |
|---|---|---|---|
| Ever use | | | |
| No | 1.00 (650, 767) | 1.00 (198, 214) | 1.00 (66, 90) |
| Yes | 1.05 (477, 541) | 1.01 (287, 303) | 1.14 (254, 303) |
| 95% CI | (0.9–1.2) | (0.8–1.3) | (0.8–1.6) |
| Years of use | | | |
| <5 | 0.95 (263, 332) | 0.82 (113, 149) | 0.98 (108, 147) |
| 5–9 | 1.05 (102, 116) | 1.15 (75, 70) | 1.18 (69, 70) |
| 10–14 | 1.30 (62, 56) | 1.16 (49, 45) | 1.64 (46, 36) |
| 15+ | 1.70[a] (42, 28) | 1.26 (45, 37) | 1.43 (29, 45) |
| Trend test | 3.91 ($P$=0.03) | 1.55 ($P$=0.11) | 3.66 ($P$=0.03) |
| Years since initial use | | | |
| <10 | 1.02 (243, 292) | 0.97 (131, 148) | 1.31 (127, 147) |
| 10–14 | 0.98 (99, 119) | 1.09 (71, 69) | 1.94[a] (62, 46) |
| 15–19 | 1.16 (60, 59) | 0.87 (31, 37) | 0.96 (33, 47) |
| 20+ | 1.33 (59, 49) | 1.09 (50, 46) | 0.68 (31, 60) |
| Trend test | 1.55 ($P$=0.11) | 0.03 ($P$=0.43) | 1.09 ($P$=0.15) |

Numbers of cases, controls are shown in parentheses. RRs are adjusted for age; RRs for years of use among the ovaries removed group are adjusted additionally for interval since oophorectomy. [a]$P<0.05$.

**Table V** Hormone-associated relative risks of breast cancer associated with years of use of menopausal hormones by selected risk factors

| | *Years of hormone use* | | |
|---|---|---|---|
| | *Nonuser* | *<10* | *10+* |
| Age (years) | | | |
| <45 | 1.00 (34) | 2.07 (28) | 0.85 (2) |
| 45–54 | 1.00 (231) | 1.08 (270) | 0.78 (32) |
| 55–64 | 1.00 (391) | 0.88 (341) | 1.45[a] (168) |
| 65+ | 1.00 (275) | 0.79 (96) | 1.43 (76) |
| Age at menarche (years) | | | |
| <12 | 1.00 (170) | 0.74 (116) | 0.66 (31) |
| 12 | 1.00 (207) | 0.80 (169) | 1.23 (75) |
| 13 | 1.00 (273) | 1.06 (220) | 1.26 (78) |
| 14 | 1.00 (152) | 1.00 (133) | 1.86[a] (54) |
| 15+ | 1.00 (118) | 1.27 (94) | 2.03[a] (38) |
| Age at first livebirth (years) | | | |
| <20 | 1.00 (63) | 1.52 (61) | 1.68 (22) |
| 20–24 | 1.00 (281) | 0.92 (228) | 1.63[a] (84) |
| 25–29 | 1.00 (248) | 0.87 (215) | 1.02 (64) |
| 30+ | 1.00 (167) | 0.67 (98) | 1.06 (46) |
| Nulliparous | 1.00 (170) | 1.22 (130) | 1.22 (59) |
| Age at menopause (years) | | | |
| <40 | 1.00 (130) | 1.02 (107) | 1.17 (67) |
| 40–44 | 1.00 (156) | 0.98 (114) | 0.99 (63) |
| 45–49 | 1.00 (252) | 0.95 (218) | 1.52[a] (76) |
| 50–54 | 1.00 (319) | 0.89 (241) | 1.76[a] (57) |
| 55+ | 1.00 (74) | 1.04 (55) | 1.41 (15) |
| History of breast biopsy | | | |
| No | 1.00 (741) | 0.96 (553) | 1.23 (199) |
| Yes | 1.00 (190) | 0.85 (182) | 1.50 (79) |
| Family history of breast cancer (first degree relative) | | | |
| No | 1.00 (687) | 0.95 (534) | 1.46[a] (219) |
| Yes | 1.00 (243) | 0.88 (197) | 0.94 (58) |

Numbers of cases are shown in parentheses. RRs are adjusted for age, type of menopause, and interval since oophorectomy. [a]$P<0.5$.

**Table VI** Relative risks of breast cancer in relation to history of benign breast disease and timing of menopausal hormone use

| *History of biopsy for benign breast disease and timing of hormone use* | *Cases* | *Controls* | *RR* | *(95% CI)* |
|---|---|---|---|---|
| Biopsy, no hormone use | 190 | 167 | 1.00 | (—) |
| Hormone use before first biopsy | | | | |
| Ever use | 48 | 70 | 0.60 | (0.4–0.9) |
| <10 years use | 29 | 40 | 0.62 | (0.4–1.1) |
| 10+ years use | 19 | 30 | 0.62 | (0.3–1.2) |
| Hormone use after first biopsy | | | | |
| Ever use | 205 | 152 | 1.14 | (0.8–1.6) |
| <10 years use | 148 | 134 | 0.93 | (0.7–1.3) |
| 10+ years use | 57 | 18 | 3.01 | (1.6–5.5) |

RRs are adjusted for age and type of menopause; RRs for years of use are adjusted additionally for interval since oophorectomy.

Associations were also examined according to whether breast cancer was diagnosed on the first screening examination (prevalent cases) as opposed to subsequently (incident cases). No differences in the associations with hormone use were apparent. However, analyses by stage of disease indicated that hormone effects predominated among the lower stage tumours (Table VII). The risks associated with ever use of hormones were 1.3 for the *in situ* tumours and 1.2 for the small invasive cancers, as opposed to 1.0 for the larger invasive tumours. Among the lower stage tumours, there were significant increases in risk with increasing duration of hormone use, with RRs rising among long-term users (10+ years) to significant risks of 1.9 and 1.5 for in situ and small invasive tumours, respectively. In comparison, the risk of large invasive tumours was 1.3 among long-term users. Menopausal hormone use was not associated with any increased risk among those tumours classified as unknown stage. The elevated risks of in situ tumours associated with long-term use were observed for all menopause subgroups, ranging from 2.1 for the naturally menopausal women to 3.0 for those having undergone a bilateral oophorectomy. In addition, increased risks associated with long-term use were observed for in situ tumours detected on the initial screening examination (prevalent cases) as well as those detected at later exams (incident cases).

**Table VII** Relative risks of breast cancer associated with menopausal hormone use by stage of disease

| | In situ (*n*=*254*) | *Small invasive* ≦*1 cm*) (*n*=*281*) | *Large invasive* (>*1 cm*) (*n*=*1110*) | *Unknown* (*n*=*315*) |
|---|---|---|---|---|
| Ever use | | | | |
| No | 1.00 (106) | 1.00 (122) | 1.00 (533) | 1.00 (170) |
| Yes | 1.26 (148) | 1.19 (159) | 1.02 (577) | 0.80 (145) |
| 95% CI | (0.9–1.6) | (0.9–1.5) | (0.9–1.2) | (0.6–1.1) |
| Years of use | | | | |
| <5 | 0.90 (57) | 0.99 (70) | 0.90 (279) | 0.79 (80) |
| 5–9 | 1.52[a] (41) | 1.40 (42) | 1.05 (138) | 0.68 (28) |
| 10+ | 1.90[a] (49) | 1.51[a] (45) | 1.29[a] (151) | 0.88 (33) |
| Trend test | 11.68 ($P<0.01$) | 5.45 ($P=0.01$) | 3.00 ($P=0.04$) | 2.04 ($P=0.08$) |

RRs are adjusted for age and type of menopause; RRs for years of use are adjusted additionally for interval since oophorectomy. [a]$P<0.05$.

### Discussion

This study found no relationship between ever use of menopausal hormones and risk of breast cancer. However, there was a significant trend in risk with increasing duration of hormone use, although elevations in risk were small, being on the order of 50% only after 15 years of use. While some further increases in risk were observed after 25 years of use, the maximum RR only reached 1.7. Thus, our findings indicate that if hormone use increases breast cancer risk, the risk is limited to relatively long-term users and is small, at least in comparison to the risks for oestrogen-related endometrial cancer (Brinton *et al.*, 1984). However, given the extensive population exposure to menopausal oestrogens and the frequency of breast cancer in the general population, even a slight excess risk associated with hormone use is cause for concern. For instance, if the relationship is causal, a 50% elevation in risk among oestrogen users of 15 or more years would result in an approximate cumulative absolute excess risk of 2% for women aged 65–79.

Of primary interest in our study was the evaluation of effect modification according to ovarian status. The issue is complicated by the fact that ovarian status influences both the risk of breast cancer (Trichopoulos *et al.*, 1972) and the rates of exposure to menopausal oestrogens (Rosenberg *et al.*, 1979). Previous studies have produced inconsistent findings. Ross *et al.* (1980) found excess hormone risks confined to women whose ovaries were left intact, particularly among those with high cumulative oestrogen doses. Hoover *et al.* (1981), however, in a study among pre-paid health plan participants, observed hormone effects to be greater in women whose ovaries were removed, a finding similar to that derived from previous analyses performed on a subset of the current data (Brinton *et al.*, 1981). In contrast, the present study found excess risks associated with long term use among all menopause subgroups, failing to support earlier impressions that women with oophorectomy are more susceptible to hormone effects than those whose ovaries are left intact. However, it must be emphasized that this conclusion is based on a careful analysis of the effects of confounding variables and particularly the interval since oophorectomy, a variable that has not been extensively evaluated in previous investigations.

Thus, it seems likely that previous findings regarding differential effects of hormones by ovarian status may reflect the influence of extraneous factors. Chance is also a possible explanation, especially if oestrogen use conveys only a modest elevation in risk, and this risk is restricted to long-term users. In the present study, which is the largest to date, we had (with 0.05 level of significance) a 90% power of detecting a RR of 1.4 among hormone users of 10 years or more. The next largest studies permitting power calculations (Hoover *et al.*, 1981; Kaufman *et al.*, 1984), however, had comparable power to detect risks in heavily exposed subjects of only 1.8 to 2.6. The ability to identify associations in subgroups, including those defined by ovarian status, would obviously be considerably less. Further complicating the interpretation of several studies (Kelsey *et al.*, 1981; Kaufman *et al.*, 1984) was the utilization of hospital controls, which may have underestimated relative risks associated with oestrogen use (Hoover *et al.*, 1978; Hulka *et al.*, 1982; Nomura *et al.*, 1986).

It was also of interest to determine whether hormone effects were altered by the presence of other risk factors, as shown in other studies, such as a family history of breast cancer (Brinton *et al.*, 1981; Hoover *et al.*, 1981; Hulka *et al.*, 1982; Nomura *et al.*, 1986), nulliparity (Brinton *et al.*, 1981), and obesity (Sherman *et al.*, 1983). Most consistently identified has been a history of benign breast disease (Hoover *et al.*, 1976; Ross *et al.*, 1980; Brinton *et al.*, 1981; Thomas *et al.*, 1982; Nomura *et al.*, 1986). This interaction seems plausible, since menopausal hormones predispose to benign breast disease (Trapido *et al.*, 1984; Berkowitz *et al.*, 1985) and benign breast disease enhances the risk of breast cancer (Ernster, 1981).

It was thus noteworthy that we found evidence of such an interaction, though it was dependent on the timing of hormone use, with all of the excess risk restricted to use after diagnosis of benign disease. In addition, the risk was further enhanced among those with extended durations of use, being at a significant 3-fold excess risk compared to those reporting no previous hormone use. A higher risk among women whose benign breast disease preceded first oestrogen use is directly opposite to that noted by Hoover *et al.* (1976). However, our findings were consistent with those of Thomas *et al.* (1982), suggesting a promotional effect of oestrogen use on benign breast pathology.

In contrast to previous reports we found no evidence of interaction with either family history of breast cancer, nulliparity, or obesity; in fact, hormone risks were somewhat elevated among these without a family history and either those with an early first birth or multiple births, possibly reflecting an enhanced ability for effects to be detected in those at lowered disease risk. Although there was some evidence of increased risk for hormone users with later ages at menopause, consistent with a common mechanistic pathway for the two factors, the interaction was not pronounced or significant.

Of special interest is our finding that hormone effects were most pronounced for the lower stage tumours. Although menopausal oestrogens have not previously been investigated in terms of breast cancer stage, Vessey *et al.* (1979) noted higher rates of oral contraceptive use among women with lower breast cancer stages, and suggested that this might reflect better diagnostic ascertainment among those with frequent contact with the health care system. However, in the present study, which focussed on a sample of volunteer participants to a screening program, ascertainment bias was of lesser concern, particularly since associations persisted for both prevalent as well as incident cases. Thus, our findings appear noteworthy, particularly given their consistency with studies that have shown that oestrogen effects are most apparent for lower grade endometrial cancers (Brinton *et al.*, 1984). Although an inverse relationship of oestrogen use with disease stage is opposite to what would be expected under a growth promotion hypothesis, it is very possible that hormones induce the proliferation of initiated cells prior to the appearance of in situ disease. Further assessment of the relationship of oestrogen use to breast cancer stage and to precursor lesions should help to clarify the carcinogenic risks and mechanisms of action.

### References

BERKOWITZ, G.S., KELSEY, J.L., HOLFORD, T.R. & 6 others (1985). Estrogen replacement therapy and fibrocystic breast disease in postmenopausal women. *Am. J. Epidemiol.*, **121**, 238.

BRINTON, L.A. (1984). The relationship of exogenous estrogens to cancer risk. *Cancer Det. Prev.*, **7**, 159.

BRINTON, L.A., HOOVER, R.N. & FRAUMENI, J.F., JR. (1983). Epidemiology of minimal breast cancer. *JAMA*, **249**, 483.

BRINTON, L.A., HOOVER, R.N., SZKLO, M. & FRAUMENI, J.F., JR. (1981). Menopausal estrogen use and risk of breast cancer. *Cancer*, **47**, 2517.

ERNSTER, V.L. (1981). The epidemiology of benign breast disease. *Epidemiol. Rev.*, **3**, 184.

HENDERSON, B.E., ROSS, R.K., PIKE, M.C. & CASAGRANDE, J.T. (1982). Endogenous hormones as a major factor in human cancer. *Cancer Res.*, **42**, 3232.

HIATT, R.A., BAWOL, R., FRIEDMAN, G.D. & HOOVER, R. (1984). Exogenous estrogen and breast cancer after bilateral oophorectomy. *Cancer*, **54**, 139.

HOOVER, R., BAIN, C., COLE, P. & MACMAHON, B. (1978). Oral contraceptive use: Association with frequency of hospitalization and chronic disease risk indicators. *Am. J. Public Health*, **68**, 335.

HOOVER, R., GLASS, A., FINKLE, W.D., AZEVEDO, D. & MILNE, K. (1981). Conjugated estrogens and breast cancer risk in women. *J. Natl Cancer Inst.*, **67**, 815.

HOOVER, R., GRAY, L.A., SR., COLE, P. & MACMAHON, B. (1976). Menopausal estrogens and breast cancer. *N. Engl. J. Med.*, **295**, 401.

HULKA, B.S., CHAMBLESS, L.E., DEUBNER, D.C. & WILKINSON, W.E. (1982). Breast cancer and estrogen replacement therapy. *Am. J. Obstet. Gynecol.*, **143**, 638.

INTERNATIONAL AGENCY FOR RESEARCH ON CANCER. IARC monographs on the evaluation of the carcinogenic risk of chemicals to humans. *Sex Hormones (II)*, Vol. 21. IARC, Lyon, 1979.

JICK, H., WALKER, A.M., WATKINS, R.N. & 6 others (1980). Replacement estrogens and breast cancer. *Am. J. Epidemiol.*, **112**, 586.

KAUFMAN, D.W., MILLER, D.R., ROSENBERG, L. & 4 others (1984). Noncontraceptive estrogen use and the risk of breast cancer. *JAMA*, **252**, 63.

KELSEY, J.L., FISCHER, D.B., HOLFORD, T.R. & 4 others (1981). Exogenous estrogens and other factors in the epidemiology of breast cancer. *J. Natl Cancer Inst.*, **67**, 327.

LUBIN, J. (1981). A computer program for the analysis of matched case-control studies. *Comput. Biomed. Res.*, **14**, 138.

NOMURA, A.M.Y., KOLONEL, L.N., HIROHATA, T. & LEE, J. (1986). The association of replacement estrogens with breast cancer. *Int. J. Cancer*, **37**, 49.

PRENTICE, R.L. & PYKE, R. (1979). Logistic disease incidence models and case-control studies. *Biometrika*, **66**, 408.

ROSENBERG, L., SHAPIRO, S., KAUFMAN, D.W., SLONE, D., MIETTINEN, O.S. & STOLLEY, P.D. (1979). Patterns and determinants of conjugated estrogen use. *Am. J. Epidemiol.*, **109**, 676.

ROSS, R.K., PAGANINI-HILL, A., GERKINS, V.R. & 4 others (1980). A case-control study of menopausal estrogen therapy and breast cancer. *JAMA*, **243**, 1635.

SHERMAN, B., WALLACE, R. & BEAN, J. (1983). Estrogen use and breast cancer. Interaction with body mass. *Cancer*, **51**, 1527.

THOMAS, D.B., PERSING, J.P., HUTCHINSON, W.B. (1982). Exogenous estrogens and other risk factors for breast cancer in women with benign breast diseases. *J. Natl Cancer Inst.*, **69**, 1017.

TRAPIDO, E.J., BRINTON, L.A., SCHAIRER, C. & HOOVER, R. (1984). Estrogen replacement therapy and benign breast disease. *J. Natl Cancer Inst.*, **73**, 1101.

TRICHOPOULOS, D., MACMAHON, B. & COLE, P. (1972). Menopause and breast cancer risk. *J. Natl Cancer Inst.*, **48**, 605.

VESSEY, M.P., DOLL, R., JONES, K., MCPHERSON, K. & YEATES, D. (1979). An epidemiological study of oral contraceptives and breast cancer. *Br. Med. J.*, **1**, 1757.